# Antibiotic resistance and metabolic profiles as functional biomarkers that accurately predict the geographic origin of city metagenomics samples

**DOI:** 10.1186/s13062-019-0246-9

**Published:** 2019-08-20

**Authors:** Carlos S. Casimiro-Soriguer, Carlos Loucera, Javier Perez Florido, Daniel López-López, Joaquin Dopazo

**Affiliations:** 1 Clinical Bioinformatics Area, Fundación Progreso y Salud (FPS), CDCA, Hospital Virgen del Rocio, c/Manuel Siurot s/n, 41013 Sevilla, Spain; 2 Functional Genomics Node (INB), FPS, Hospital Virgen del Rocio, 41013 Sevilla, Spain; 3 Bioinformatics in Rare Diseases (BiER), Centro de Investigación Biomédica en Red de Enfermedades Raras (CIBERER). FPS, Hospital Virgen del Rocio, 41013 Sevilla, Spain

**Keywords:** Machine learning, Classification, Metagenomics, Whole genome sequencing, Functional profiling, Antibiotic resistance

## Abstract

**Background:**

The availability of hundreds of city microbiome profiles allows the development of increasingly accurate predictors of the origin of a sample based on its microbiota composition. Typical microbiome studies involve the analysis of bacterial abundance profiles.

**Results:**

Here we use a transformation of the conventional bacterial strain or gene abundance profiles to functional profiles that account for bacterial metabolism and other cell functionalities. These profiles are used as features for city classification in a machine learning algorithm that allows the extraction of the most relevant features for the classification.

**Conclusions:**

We demonstrate here that the use of functional profiles not only predict accurately the most likely origin of a sample but also to provide an interesting functional point of view of the biogeography of the microbiota. Interestingly, we show how cities can be classified based on the observed profile of antibiotic resistances.

**Reviewers:**

Open peer review: Reviewed by Jin Zhuang Dou, Jing Zhou, Torsten Semmler and Eran Elhaik.

## Background

In recent years there has been an increasing interest in microbiome research, especially in the context of human health [1–4]. However, bacteria are ubiquitous and microbiotas from many different sources have been object of scrutiny [5]. Specifically, environmental metagenomics of soil and oceans is gaining much attention [6–10]. However, urban environments have comparatively received less less and only a few reports on urban microbial communities have been published [11–13]. The Metagenomics and Metadesign of the Subways and Urban Biomes (MetaSUB) is an International Consortium with a wide range of aims, currently involved in the detection, measurement, and design of metagenomics within urban environments [14]. Typically, microbiomes have been studied by analyzing microbial abundance profiles obtained either from 16S RNAs or from whole genome sequencing (WGS), which can be further related to specific conditions [15, 16]. More recently, 16sRNA data has been used as a proxy to derive functional profiles by assigning to each sample the functional properties (pathways, resistance or virulence genes, etc.) of the genomes of reference of each species identified in it [17, 18]. However, 16sRNA data does not allow direct inference of genes actually present in the bacterial population studied [19]. Contrarily, metagenomics shotgun sequencing allows inferring a quite accurate representation of the real gene composition in the bacterial pool of each sample that can be used to identify strain-specific genomic traits [20, 21]. For example, the focused study of specific traits such as antibiotic resistance or virulence genes has been used to detect pathogenic species among commensal strains of *E. coli* [22]. Also, general descriptive functional profile landscapes have been used to understand the contribution of microbiota to human health and disease [22–24]. Moreover, another aspect of crucial interest is the use of microbiota in forensics [25]. Microbial communities differ in composition and function across different geographical locations [25], even at the levels of different cities [26–28]. Thus, data on specific microbiomes composition in a host or environment can help in determining its geographic location [26]. However, the value of existing functional profiling tools when applied to environmental microbiota and, specifically, to urban metagenomes, that can provide an extra perspective of biological interpretation, remains to be explored.

Here, we propose a machine learning innovative approach in which functional profiles of microbiota samples, obtained from shotgun sequencing, are used as features for predicting geographic origin. Moreover, in the prediction schema proposed, a feature relevance method allows extracting the most important functional features that account for the classification. Thus, any sample is described as a collection of functional modules (e.g. KEGG pathways, resistance genes, etc.) contributed by the different bacterial species present in it, which account for potential metabolic and other functional activities that the bacterial population, as a whole, can perform. We show that the functional profiles, obtained from the individual contribution of each bacterial strain in the sample, not only display a high level of predictive power to detect the city of origin of a sample but also provide an interesting functional perspective of the city analyzed. Interestingly, relevant features, such as antibiotic resistances, can accurately predict the origin of samples and are compatible with epidemiological and genetic observations.

## Material and methods

### Data

Sequence data were downloaded from the CAMDA web page (http://camda2018.bioinf.jku.at/doku.php/contest_dataset#metasub_forensics_challenge). There are four datasets: *training dataset* composed of 311 samples from eight cities (Auckland, Hamilton, New York, Ofa, Porto, Sacramento, Santiago and Tokyo), *test dataset 1*, containing 30 samples from New York, Ofa, Porto and Santiago; *test dataset 2* containing 30 samples from three new cities (Ilorin, Boston and Lisbon) and *test dataset 3* containing 16 samples from Ilorin, Boston and Bogota.

### Sequence data processing

**Fig. 1:**
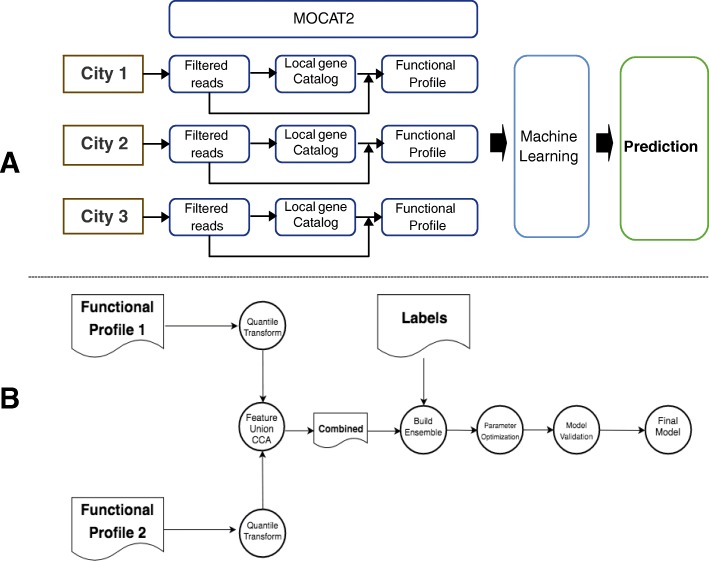
Schemas of: **a** The annotation and machine learning procedure and **b** The fusion pipeline, as explained in Methods

Local functional profiles were generated from the original sequencing reads by the application MOCAT2 [29] which uses several applications for the different steps. FastX toolkit is used for trimming the reads and SolexaQA [30] to keep the reads in which all quality scores are above 20 and with a minimum length of 45. In order to remove possible contamination with human genomes we screened the reads against hg19. In this step MOCAT2 use SOAPaligner v2.21 [31]. High quality reads were assembled with SOAPdenovo v1.05/v1.06 [31]. Then, genes were detected inside contigs using Prodigal [32]. Figure 1a outlines the procedure followed.

### Functional profiles

CD-HIT software [33] with a 95% identity and a of 90% overlap with the sorter sequence was used to create a local gene catalog for each city. Gene catalogs were annotated using DIAMOND (v0.7.9.58) [34] to align the genes against the orthologues groups of the database eggNOG (v4.5) [35]. MOCAT2 pre-computed eggNOG orthologous groups sequences with annotations from other databases. Then, a functional profile is generated for each sample by assessing the gene coverage for KEGG (v74/57) [36] and CARD (August 2015) [37] functional modules. Finally, each sample is normalized by the number of mapped reads against local gene catalog.

### Machine learning pipeline

The machine learning phase takes the complete KEGG Module functional profile as the input feature space, i.e. each training/validation sample is represented as a 1D-array where the values/features are a one to one map with the KEGG modules. The machine learning pipeline has been implemented in python 3.6 by making use of scikit-learn [38]. The training and validation datasets are transformed according to a quantile transformation whose parameters are learned from the training data. Subsequently, we apply the learned data representation to each validation dataset. The quantile preprocessing performs a feature-wise non-linear transformation which consists on transforming each variable to follow a normal distribution. This is a robust preprocessing scheme since the impact of the outliers is minimized by spreading the most frequent values.

In order to visualize such a high dimensional dataset we use the t-distributed Stochastic Neighbor Embedding (t-SNE) [39] methodology. Due to the fact that the feature space dimension is much greater than the number of samples, a principal component analysis (PCA) is performed to reduce the dimensionality of the embedding process carried out by t-SNE.

### Classification pipeline

To classify each sample into one of the known cities a classification pipeline was developed which mainly consists of: i) A base learner with decision trees, ii) An ensemble of base learners via Scalable Tree Boosting [40] and, iii) A Bayesian optimization framework for tuning the hyper parameters. The optimization tuning has been done by following the guidelines provided in [41]. We chose to use here Scalable Tree Boosting Machine learning because of its proven performance in other similar problems involving multi-view scenarios and because of its easy interpretability [42].

In order to estimate the generalization error of the underlying model and its hyper-parameter search we have used a nested/non-nested cross-validation scheme. On the one hand, the non-nested loop is used to learn an optimized set of hyper-parameters, on the other hand, the nested loop is used to estimate the generalization error by averaging test set scores over several dataset splits. The scoring metric is the accuracy and the hyper-parameter learning is done on the inner/nested cross validation by means of Bayesian optimization. Figure 1a contains a schema of the whole pipeline followed here.

### Fusion pipeline

In order to improve the classification accuracy of the proposed method we can fuse different functional profiles by learning an approximation of the latent space by means of Canonical Correlation Analysis (CCA) and then applying the machine learning pipeline already proposed. Thus, a multi view classification problem, where the views are the functional profiles can be constructed. A quantile transformation is learned for each dataset as previously described (Fig. 1a) and then, the latent space between both views is built by making use of CCA as previously described [43]. Finally, we apply the proposed classification pipeline (except the quantile transformation).

Given two datasets X_1_ and X_2_ that describe the same samples (two views of the samples), CCA-based feature fusion consists in concatenating, or adding, the latent representations of both views in order to build a single dataset that captures the most relevant patterns. CCA finds one transformation (T_i_) for each view (here we have two views: KEGG and CARD, although the procedure can be generalized to incorporate more views) in such a way that the linear correlation between their projections is maximized in a latent space with less features that either X_1_ or X_2_. Figure 1b shows a diagram that summarizes the Fusion Pipeline.

## Results and discussion

### Classification of the cities

The CAMDA challenge *test dataset* consists of 311 samples from eight cities: Auckland, Hamilton, New York, Ofa, Porto, Sacramento, Santiago and Tokyo. The predictor was trained with this test dataset and then used to predict new samples.

**Fig. 2:**
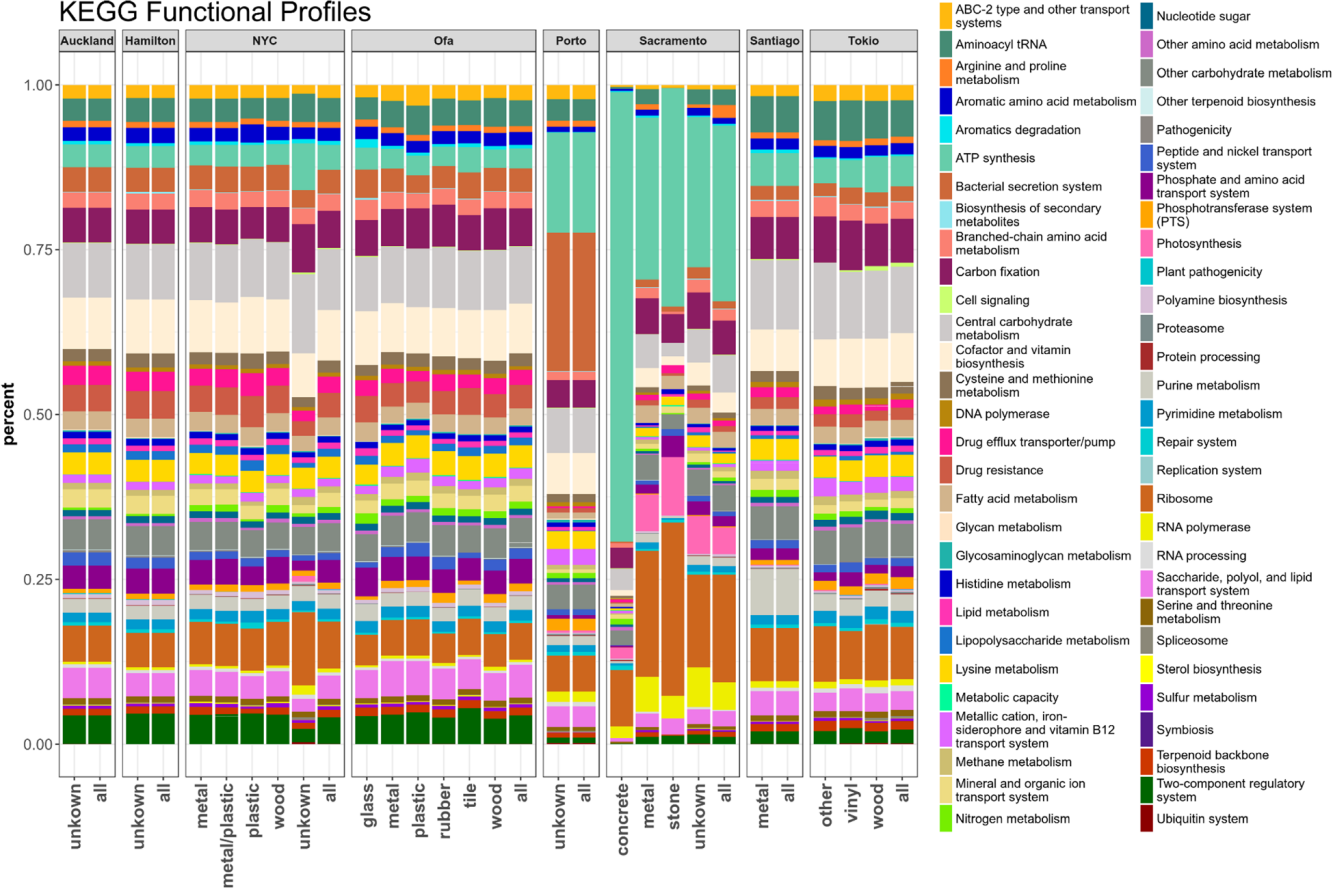
Percentages of 59 high level KEGG modules defining the functional profiles for each city and surface by city are shown (for the sake of the visualization KEGG modules were collapsed to the corresponding highest-level definitions)

**Fig. 3:**
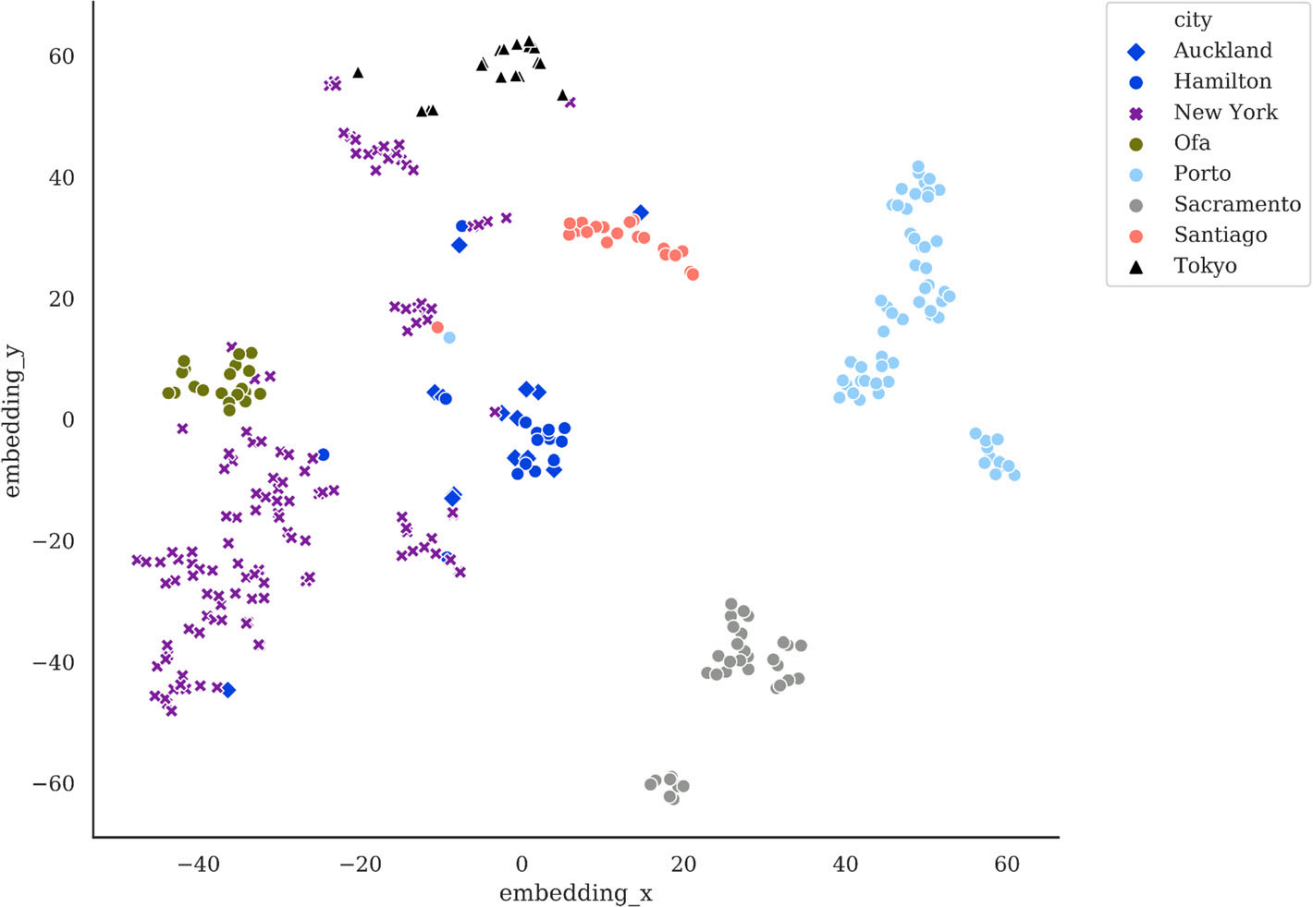
Classification of the cities of the training set based on KEGG-based functional profiles using a(t-SNE) [39] plot. As expected, the New York cluster shows the highest dispersion. Hamilton and Auckland (both New Zealand cities connected by a train) are separated from the other cities but are very difficult to distinguish among them

The sequences from the CAMDA *test dataset* were processed as described in methods and a KEGG-based functional profile was obtained for all the samples of the training datasets. We observed that local catalog size was highly city-dependent (Auckland: 293,210; Hamilton: 472,649; NYC: 1,147,284; Ofa: 1,397,333; Porto: 76,083; Sacramento: 65,120; Santiago: 168,523; Tokyo: 449634). Also, the degree of contamination by reads identified as humans fluctuated across cities (Auckland: 278,183; Hamilton: 340,532; NYC: 227,888,129; Ofa: 410,909; Porto: 107,053,017; Sacramento: 40,028,005; Santiago: 158,313,417; Tokyo: 515,448,367). The cities display characteristic functional profiles (see Fig. 2) that clearly differentiate them. Figure 3 shows how the functional profiles separate the different cities as result of the application of the clustering pipeline on the *training dataset 1*. The results reveal the strong performance of the suggested pipeline as most of the classes (i.e. cities) are well separated, with the exception of Hamilton and Auckland (both New Zealand cities) which are clearly differentiated from the other cities but map together, as the train line sampled links both cities. This functional similarity was expected due to their geographical closeness and its connection. Table 1 shows the cross-validation results, where the New Zealand cities could not be properly resolved as some of the samples were miss assigned.

**Table 1:** Cross validation of the CAMDA training dataset

Truth / Pred	Auckland	Hamilton	NY	Ofa	Porto	Sacramento	Santiago	Tokyo	All
Auckland	9	4	0	1	0	1	0	0	15
Hamilton	3	11	2	0	0	0	0	0	16
NY	1	0	110	1	0	6	2	6	126
Ofa	0	0	3	17	0	0	0	0	20
Porto	0	0	0	0	60	0	0	0	60
Sacramento	0	0	0	0	0	34	0	0	34
Santiago	0	0	1	0	0	0	17	2	20
Tokyo	0	0	0	0	0	0	0	20	20
All	13	15	116	19	60	41	19	28	311

### Feature extraction and biological relevance in the classification

An advantage of using functional modules as classification features is that their biological interpretation is straightforward. Here, the most relevant features were extracted from the classification pipeline from each run of the experiment, cross referencing the nested loop for the best set of hyperparameters and a final fit with all training data, by averaging the feature importance of each base learner of the ensemble. The features that appeared in all the experiments were selected. Then, to assure the relevance of each extracted feature we cross-reference it with those found by an l1-driven logistic regression model. Finally, we perform a 10-fold cross-validated prediction in order to assess that the difference in accuracy is close to that found with the whole dataset. The total number of extracted features adds up to 44.

**Fig. 4:**
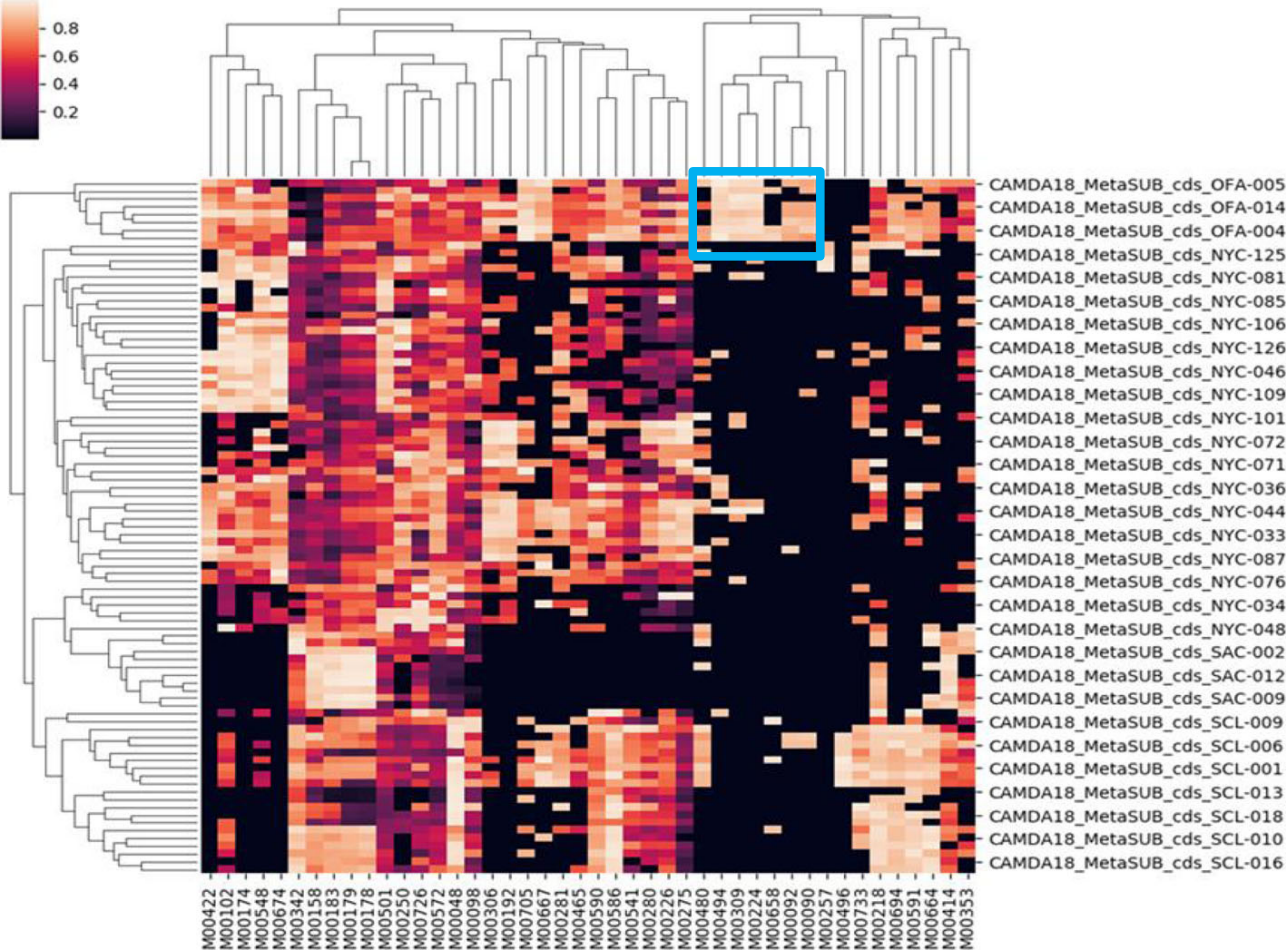
The most relevant KEGG features extracted from the classification pipeline by averaging the feature importance of each base learner of the ensemble in each run of the experiment. In a blue square the features characteristic from Ofa, and listed in Table 2, are shown

Importantly, the features used for the classification have a direct biological meaning and account for city-specific functional properties of the bacterial samples found in each city. As an example of easy interpretation is the city of Ofa. Out of the seven most relevant features that distinguish this city from the rest of cities (see Fig. 4), three KEGG modules are related with antibiotic resistances (see Table 2). Interestingly, antibiotic resistance had already been studied in the MetSUB dataset by directly searching the presence in *P. stutzeri mexA* strains (that carry the *mexA* gene, a component of the MexAB-OprM efflux system, that confer resistance to antibiotics [44]) present in samples from some cities [13]. However, in the approach presented here, that allowed the detection of the most relevant functional features that characterize cities, antibiotic resistance arises as a highly discriminative feature for some of them.

**Table 2:** The most relevant KEGG modules in Ofa

KEGG ID	KEGG name
M00090	Phosphatidylcholine (PC) biosynthesis, choline = > PC
M00092	Phosphatidylethanolamine (PE) biosynthesis, ethanolamine = > PE
M00224	Fluoroquinolone transport system
M00309	Non-phosphorylative Entner-Doudoroff pathway, gluconate/galactonate = > glycerate
M00480	VraS-VraR (cell-wall peptidoglycan synthesis) two-component regulatory system
M00494	NatK-NatR (sodium extrusion) two-component regulatory system
M00658	VanS-VanR (actinomycete type vancomycin resistance) two-component regulatory system

Particularly, the *Fluoroquinolone transport system* (M00224) is an ABC-2 type transporter that confers resistance to fluoroquinolone, a widely used antibiotic [45, 46]. Similarly, *VraS-VraR* (M00480) and *VanS-VanR* (M00658) are two-component regulatory systems involved in the response to two antibiotics, β-lactam [47] and glycopeptides [48], respectively. Interestingly, *Fluoroquinolone transport system* and *VraS-VraR* are known to confer resistance in *Staphylococcus aureus*, a pathogen of recognized higher incidence rates in sub Saharan Africa than those reported from developed countries [49]. Since *Staphylococcus aureus* is a skin pathogen it is easier to find it over-represented in the African MetaSUB samples. This observation captured by the functional analysis of MetaSUB samples proposed here suggests an excessive use of antibiotics that could eventually have caused an emergence of resistant strains. Actually, epidemiologic studies report the prevalence of Staphylococcal disease in sub-Saharan Africa, along with an increase in antibiotic resistance [49]. Moreover, two single-nucleotide polymorphisms (SNPs) in the human leukocyte antigen (HLA) class II region on chromosome 6 was demonstrated to be associated with susceptibility to *S. aureus* infection at a genome-wide significant level [50]. Additionally, a recent admixture mapping study demonstrated that genomic variations with different frequencies in these SNPs in European and African ancestral genomes influence susceptibility to *S. aureus* infection, strongly suggesting a genetic basis for our observations [51].

### Classification of new samples of the cities in the training set

In order to test the prediction power of the predictor obtained using the *training dataset*, we have used the *test dataset 1* composed of 30 samples belonging to the same cities that are in the *training dataset*. Table 3 shows the cross validation and the confusion matrix, in which, the functional heterogeneity of New York clearly introduces some noise in the classification (probably with a real biological meaning). The accuracy of the predictor is of 0.73.

**Table 3:** Cross validation and confusion matrix of KEGG functional profiles obtained from the samples from the test dataset 1, belonging to the cities from the training dataset

Truth / Preds	Auckland	Hamilton	NY	Ofa	Porto	Sacramento	Santiago	Tokyo	All	Accuracy
NY	1	1	8	0	0	0	0	0	10	0.8
Ofa	0	0	2	3	0	0	0	0	5	0.6
Porto	0	0	1	0	8	0	0	1	10	0.8
Santiago	0	0	1	0	0	1	3	0	5	0.6
All	1	1	12	3	8	1	3	1	30	0.73

### Classification using different functional profiles

**Fig. 5:**
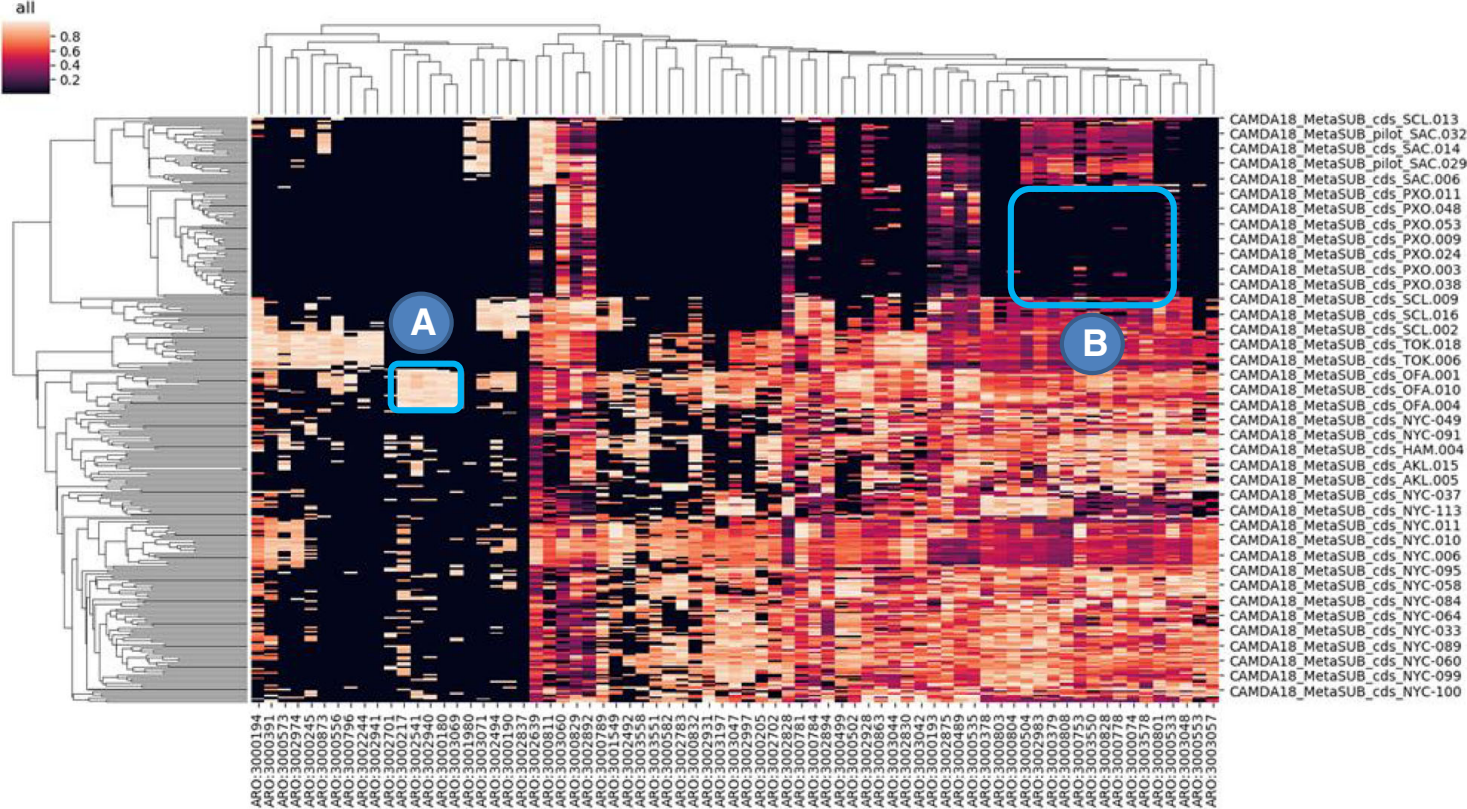
The most relevant CARD (antibiotic resistances) features extracted from the classification pipeline by averaging the feature importance of each base learner of the ensemble in each run of the experiment. **a** Features characteristic from Ofa. **b** Features characteristic from Porto

KEGG encompasses a global compendium of bacterial functionalities, providing features with a high discriminatory power. However, many KEGG modules represent too general functionalities that can be interesting for hypothesis-free discovery studies but they can mask specific modules which are relevant for more focused medical, forensic or epidemiological studies. Instead, other databases that collect specific bacterial activities or functionalities could be used. Since antibiotic resistance has emerged among the generic functionalities as a high relevant feature in the classification, in addition to have an obvious importance by itself, it seemed worth focusing on features that specifically describe antibiotic resistances. Therefore, a new training process was carried out using CARD, the database of antibiotic resistances [37]. Again, a set of antibiotic resistance features clearly distinguishes Ofa from the rest of cities, as previously observed (Fig. 5a). Table 4 describes the specific resistances distinctive of Ofa which, overall, reinforce our previous finding with KEGG about transporters [45, 46] and two-component regulatory systems involved in the response to antibiotics [47, 48], but providing more detail on specific resistance mechanisms. Interestingly, the characteristic that distinguishes Porto samples from those of other cities is the absence of antibiotic resistances (Fig. 5b). Although we do not have a strong epidemiological explanation for this, recent studies show that Portugal is among the countries in Europe with the highest defined daily antibiotic dose per habitant [52]. Whether the high antibiotic consumption is behind this observation or not needs of deeper epidemiological studies but, in any case, this result points to a distinctive local characteristic of clear epidemiological relevance.

**Table 4:** The most relevant antibiotic resistance modules (CARD) in Ofa

ACCESSION	NAME	DESCRIPTION
3002940	vanSN	vanSN is a vanS variant found in the vanN gene cluster
3000217	blaR1	blaR1 is a transmembrane spanning and signal transducing protein which in response to interaction with beta-lactam antibiotics results in upregulation of the blaZ/blaR1/blaI operon.
3003069	vanXYG	vanXYG is a vanXY variant found in the vanG gene cluster
3000180	tetA(P)	TetA(P) is a inner membrane tetracycline efflux protein found on the same operon as the ribosomal protection protein TetB(P). It is found in Clostridium, a Gram-positive bacterium.
3002541	AAC(3)-VIIa	AAC(3)-VIIa is a chromosomal-encoded aminoglycoside acetyltransferase in Streptomyces rimosus

Table 5 shows the cross validation and the confusion matrix with the CARD functional profiles, in which, the functional heterogeneity of New York is still introducing some noise in the classification but the accuracy of the predictor increased to 0.8.

**Table 5:** Cross validation and confusion matrix of antibiotic resistances (CARD) functional profiles obtained from the samples from the test dataset 1, belonging to the cities from the training dataset

Truth/pred	Auckland	NY	Ofa	Porto	Santiago	All	Accuracy
NY	2	8	0	0	0	10	0.8
Ofa	0	1	4	0	0	5	0.8
Porto	0	0	0	10	0	10	1
Santiago	0	2	0	1	2	5	0.4
All	2	11	4	11	2	30	0.8

### Classification using mixed functional profiles

In addition to build predictors with a single functional feature, it is possible to combine different functional profiles to produce higher accuracy in the classification. Here, we combined KEGG and CARD profiles using the Fusion Pipeline (see Methods) and the resulting classification accuracy increased to 0.9. Table 6 shows the cross-validation values obtained with the mixed profiles. Only New York, which is the most heterogeneous cite from a functional point of view, shows a couple of bad predictions (the Ofa misplaced sample was assigned to New York, probably for the same reason).

**Table 6:** Cross validation and confusion matrix of functional profiles obtained from the combination of KEGG and CARD corresponding to samples from the test dataset 1 belonging to the cities from the training dataset

Truth/pred	Auckland	NY	Ofa	Porto	Santiago	All	Accuracy
NY	1	8	1	0	0	10	0.8
Ofa	0	1	4	0	0	5	0.8
Porto	0	0	0	10	0	10	1
Santiago	0	0	0	0	5	5	1
All	1	11	3	10	5	30	0.9

More functional profiles could be included by using an extension of the Fusion Pipeline to N datasets as previously shown [53], coupled with robust Least Squares techniques [54], to accommodate for the challenging low sample size high dimensional data scenario.

### Classification new samples of with new cities

In order to check the performance of the predictor with samples from cities that were not used in the initial *training dataset* we used the 30 samples from the *test dataset 2*, from the cities: Ilorin (close to Ofa), Lisbon (in Portugal, but not close to Porto) and Boston (in USA, but not close to New York).

**Fig. 6:**
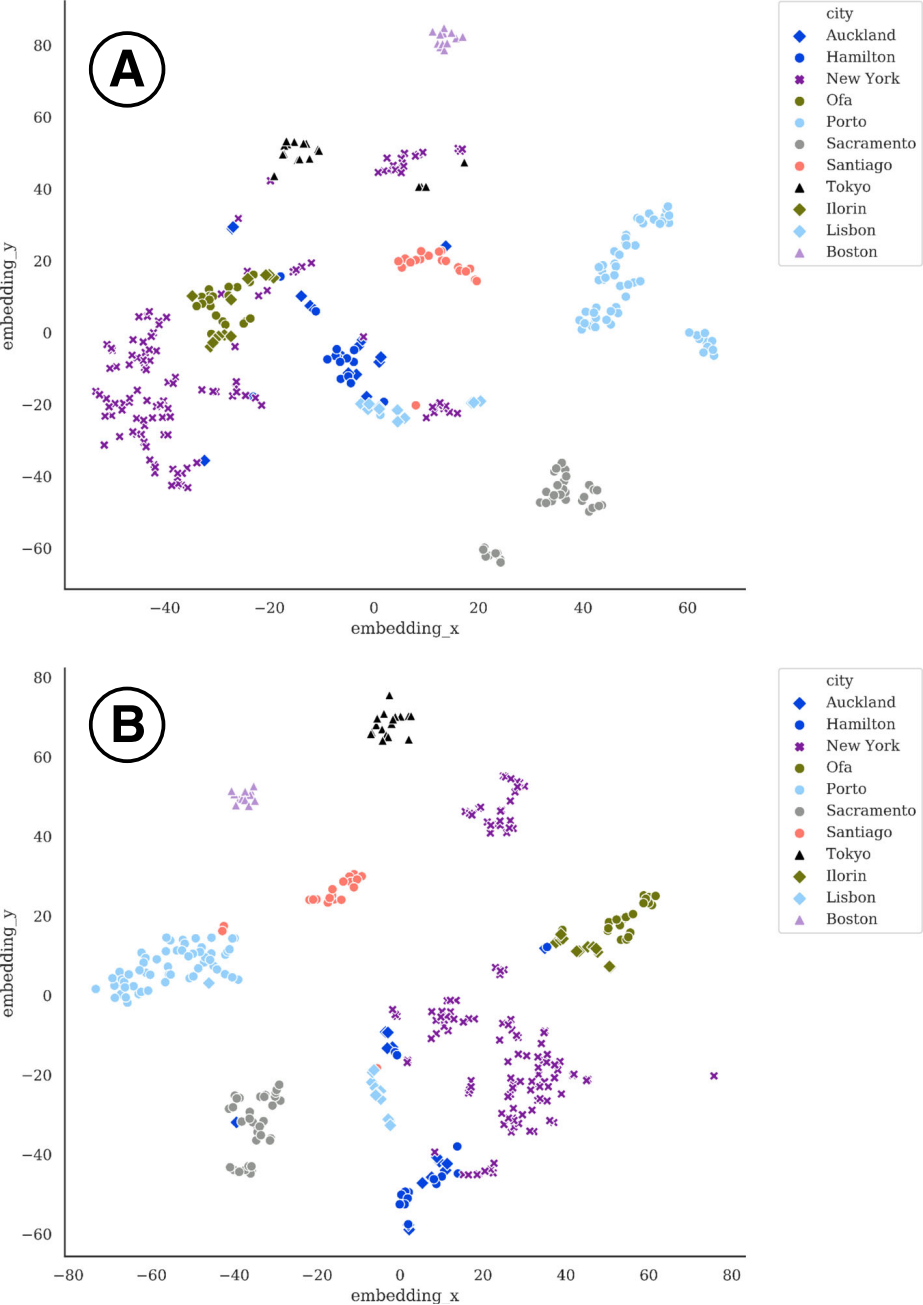
Classification of all the cities obtained with **a** KEGG-based functional profiles and **b** CARD-based functional profiles using a(t-SNE) [39] plot. Ilorin and Ofa, two physically close cities in Nigeria (connected by a train) map close to each other. New York, not close to Boston, and Lisbon, not close to Porto cluster apart in the plot. Hamilton and Auckland, both New Zealand cities connected by a train, also map together

Figure 6 shows the samples clustered in cities, as expected. Thus, Ilorin and Ofa map together because these two cities are physically close cities in Nigeria (and connected by a train). As expected, the New York cluster shows the highest dispersion. However, is does not cluster together with Boston. The same is observed with Lisbon, which is not close to Porto and both map in different places. Interestingly, the Porto “outlier” sample maps on the Lisbon cluster. Similar to the case of Ofa and Ilorin, Hamilton and Auckland, both New Zealand cities connected by a train also map together as well.

### Machine learning pipeline comparison

**Fig. 7:**
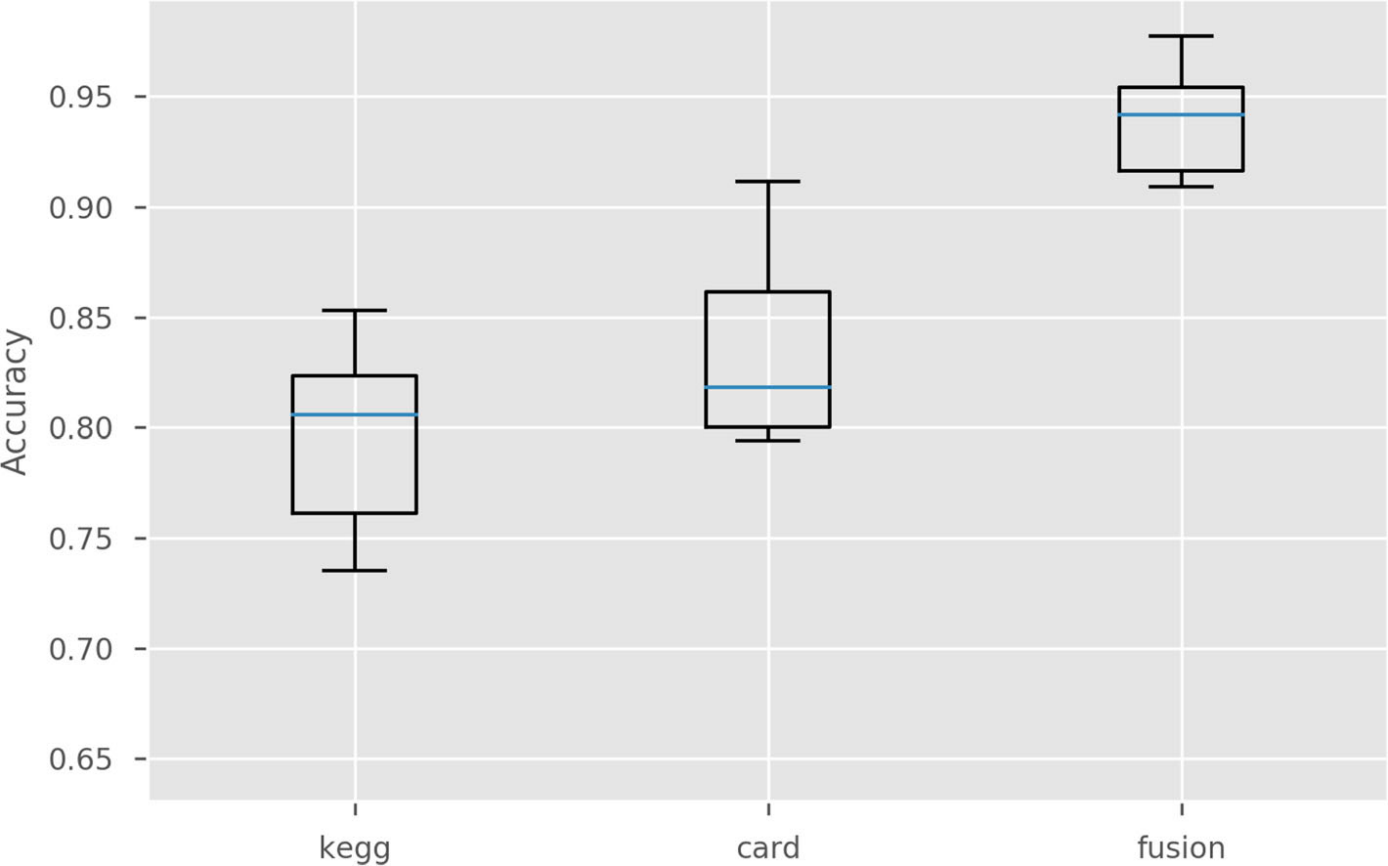
Accuracies obtained using the whole dataset (*Training dataset* and *test datasets 1, 2* and *3*) with only KEGG profiles, only CARD profiles and the fusion of both profiles

Finally, the performance of each machine learning pipeline was evaluated by joining the samples from the training and the three validation datasets. For each model a 10-fold city-wise stratified cross-validation was performed. In order to provide statistical evidence for the results each experiment is repeated 10 times with different random seeds initializations. Figure 7 shows a box plot diagram of the different experiments grouped by the functional profile used, namely: *kegg* for KEGG-Modules, *card* for CARD-ARO and *fusion* for the Multiview case. As expected, the model performance follows the tendency already exhibited: the fusion pipeline outperforms the single-view case, and the CARD-ARO *view* provides slightly better results than KEGG-Modules.

## Conclusions

The recodification of metagenomics data from the conventional gene or strain abundance profiles to other types of profiles with biological meaning offers new avenues for the analysis of microbiome data. Here we show how the use of KEGG- and CARD-based functional profiles, derived from the original metagenomics data, not only provides accurate sample classification but also offers interesting epidemiological and biological interpretations of the results found. Interestingly, antibiotic resistance arises as a relevant classification feature, supported by epidemiological [49] and genetic [51] previous observations.

## Reviewers’ comments

### Reviewer’s report 1: Jin Zhuang Dou

This paper uses transformed functional profiles from metagenomics as features for geographic origin prediction, and also provides interesting epidemiological and biological interpretations based on these features. They have also demonstrated that the proposed fusion module outperforms the single KEGG/CARD module. I think that this is a worthwhile analysis that provides a new avenue for the analysis of urban microbiome data. Their findings are just as important and viewing the purposes of Biology Direct. However, there are several points that the authors should at least consider addressing to improve the paper.

Major comments

1) L45–46 in Page3. The authors claim that “little is known on the value of existing profiling tools when applied to urban metagenomes [15]”. However, Zolfo et al. has shown that “strain-level methods developed primarily for the analysis of human microbiomes can be effective for city-associated microbiomes”. Indeed, Zolfo et al. are aimed to address the issue by testing the currently available metagenomic profiling tools on urban metagenomics. Therefore, I think the citation here is a little misleading.

Author’s response: *actually, we meant the functional profiles. We apologize for the way the sentence was written: it was a bit ambiguous. We have rewritten the sentence for clarity. We have cited Zolfo as response to point 2, as part of the background on the characterization of microbiota in urban environments.*

2) L48 in Page3. The authors do not have any introductions about the fields of predicting geographic origin from metagenomics. If no studies have involved in this topic before, the author should explain why predicting geographic origin is important for scientific communities. This will definitely improve the novelty of this work. If there are previous studies in this topic, the authors should present basic descriptions to readers who are not familiar with that. In this case, it would be interesting to see the other approaches compared/discussed in this study.

Author’s response: *we have included some background on studies of urban metagenomes. But, to our knowledge, there are no previous reports on the use of microbiota to detect the origin of a sample. We have included this information in the text as requested by the referee.*

3) L17–18 in Page4. The authors have removed reads from human genome. It will be appreciated if authors can list how many reads are from human genome.

Author’s response: *We have included in the results section, “Classification of the cities” subsection, the details requested.*

4) L24–25 in Page4. After clustering using CD-hit, how many genes are included in a local gene catalog for each city? It will be appreciated if authors can provide these details.

Author’s response: *We have included in the results section, “Classification of the cities” subsection, the details requested.*

5) L3–13 in Page6. The authors presented an example of easy interpretation for city of Ofa in Fig. 4. It is not comprehensive to only show one point here. As for me, M00496, M00733, M00218, M00694, M00733, M00591, M00664 could separate OFA and SCL from other locations. Are there any biological interpretations for this? Also, why SAC location only has M00342, M00158, M00183, M00179, M00178, M00501, M00218, and M00414?

Author’s response: *We just wanted to show an example of interpretation. Actually, a detailed biological interpretation of the observations is beyond the scope of the manuscript, which focuses on the validation of the use of functional profiles for geographical classification purposes. In any case, from the figure, the only M00694 (cGMP signaling), is shared between OFA and SLC and is absent in the rest of cities, and it is a too general module to offer an interesting biological interpretation. Regarding the rest of modules mentioned, these are either shared by other cities (M00733, M00218, M00591, M00664) or absent in OFA (M00496). With respect to the modules that define SAC, these are the ones selected by relevance in the classification by the algorithm. There are modules with very general functionalities (Ribosome, RNA polymerase, etc.), that are shared with many other cities. Al often happens in classification problems with some of the entities involved is that, the characteristic of SAC is the absence of a number of modules that are relevant for other locations.*

6) L27–42 in Page7. In Fig. 6, only KEGG-based functional profiles are presented here. In this work, authors have demonstrated that the fusion pipeline has the best performance. It is better to show the predictions from KEGG profiles, CARD profiles and the fusion of both profiles separately in Fig. 6. In addition, the embedding dimension 0 and 2 are shown. I am wondering why authors skip dimension 1? At least for me, this should be specified.

Author’s response: *We have included KEGG and CARD profiles in* Fig. 6. *While KEGG and CARD profiles show the predictive performance of the method, trained with the training datasets, the fusion has been made using all the data and obviously will cluster all the cities better. Therefore, it does not make much sense to show it. Regarding the numbering of the dimensions it was an error. There were two dimensions that should be 1 and 2. We have substituted it by X and Y for the shake of clarity.*

Minor issues

1) L8–9 in Page3. There should be only one dot at the end of this sentence.

2) L5–7 in Page4. A left parenthesis has been entered without a closing right parenthesis.

3) L9–10 in Page4. There should be one dot at the end of this sentence.

Author’s response: *All the typos have been corrected*.

4) L23–23 in Page5. It is better to add the range of i, for example, Ti, i = 1,2.

Author’s response: *The i makes reference to the number of views (here KEGG and CART). We have clarified this in the text.*

5) L41–42 in Page5. What do “TBP” mean at the bottom of Fig. 2? There is no any information about this label. The authors should add more about that in the figure legend. The current resolution of this figure is very low for a review.

Author’s response: *TBP (to be provided) refers to an unknown surface whose nature was never provided in the metadata. In any case, surfaces are irrelevant within the goal of the manuscript. We have changed TBP by unknown in the figure. We have increased the resolution of the figure as well as the size of the labels.*

### Reviewer’s report 2: Jing Zhou

In this paper, the authors predicted the geographic origin of samples from the CAMDA challenge using metabolic profiles as training features. It is very interesting that using antibiotic resistance feature only can distinguish cities as well. They also compared three machine learning pipelines, i.e. using KEGG profile only, using CARD profile only, and the combination of the two profiles. They found out the “fusion” pipeline yielded the best results among the three. This manuscript is very clear and well written. It provides both biological and technical insights into classification cities based on their metagenomics data. I believe this paper fits the standard of Biology Direct and should publish with the following comments addressed.

I wonder if the authors have compared different machine learning algorithms? Could you explain why choosing decision tree as the training algorithm?

Author’s response: *Actually, we always compare the performance of the chosen algorithm with respect to generalized linear models that were clearly outperformed by xgBoost. Moreover, this ML algorithm is one of the top winners in Kaggle contests (**https://www.kdnuggets.com/2017/10/xgboost-top-machine-learning-method-kaggle-explained.html**). We have added a sentence justifying the use of Scalable Tree Boosting Machine learning in this work.*

Minor:

1) Page 7, line 32: misspelling. “Ney York” should be “New York”.

2) The font for Table 3 looks smaller than Table 5. Please make sure the fort is consistent throughout the paper.

3) Fig. 3, the two circles in Fig. 3 are confusing. I understand the authors wanted to indicate New York and Auckland/Hamilton data points using the circles. However, the circles did not include all the data points. It’s not very accurate. Maybe just delete the circles and refer them by their colors.

Author’s response: *Misspelling has been corrected and table fonts have been homogenized. As suggested by the referee, the circles were removed in* Fig. 3 *and, for homogeneity, also in* Fig. 6.

### Reviewer’s report 3: Torsten Semmler

In their manuscript entitled “Antibiotic resistance and metabolic profiles as functional biomarkers that accurately predict the geographic origin of city metagenomics samples” Casimiro-Soriguer et al. compare the composition of metagenomics samples from different cities based on specific functional profiles obtained by matching against KEGG and CARD databases. The results gained here were then used to classify unknown samples regarding their city of origin by a machine learning approach. It is interesting to see that the markers that are more involved in the biological processes, especially those related to antimicrobial resistances are specific enough in their composition to clearly distinguish their city of origin.

Reviewer recommendations to authors:

The analyses and conclusions are sound but there are several grammar and spelling mistakes. If these would be corrected I recommend this manuscript without any doubts for publication in Biology Direct.

Author’s response: *We appreciate very much the positive comments of the referee. We have reviewed carefully the text and corrected grammar and spelling mistakes.*

### Reviewer’s report 4: Eran Elhaik

Casimiro-Soriguer and colleagues proposed to use the functional profiles that account for bacterial metabolism and other cell functionalities to classify bacteria, sampled as part of the MetaSUB consortium and made available as part of the CAMDA challenge, into the cities from which they were collected from using a machine learning algorithm. They claim that their method accurately predicts the sampling site and provides insights about the relationships of geography and function. This is an interesting approach, but much more clarity and validation are necessary. I found the manuscript quite confusing, the analyses incoherent, incomplete, and misleading and the English poor.

Author’s response: *We regret that the referee has found the “manuscript confusing, the analysis incoherent, incomplete and misleading”. It sounds a quite radical comment when the other three referees saw no major issues with the manuscript and this referee do not seem to be very familiar with ML and with the methods used here, given that he describes some terms of common use in ML as buzzwords. Moreover, a more careful reading of the manuscript can directly solve a number of issues he raised. Fortunately, the referee finds the method “interesting” as well, and we will focus on this positive impression.*

Major comments

• The “Machine learning pipeline” section is unclear. How do you make geographical predictions? It seems that the ML can only classify samples to cities. So, classification to new cities would be impossible. Is this correct? If so, this is a classification, not prediction algorithm, in which case you should not make claims about predictions and be very clear about the limitation of your approach.

Author’s response: *This is a matter of semantics. Prediction is more generic than classification. Classification of new cities is impossible without a highly detailed geographic sampling. The predictor can only give a probability of class membership for known classes. However, what is obvious from our results is that unknown cities close to known cities actually cluster together, while distant new cities appear as independent groups in the plot. Moreover,* Fig. 7 *suggests that, the more geographical points are added the better is the classification, which supports that a detailed geographical sampling would actually convert the predictor into a city classifier.*

• Figure 2, did you use the sampling material for the algorithm? If so, why present it? If you don’t even discuss it. Either discuss the materials or removed this figure.

Author’s response: *This figure is mentioned in results as a visual differentiation among cities based on average functional profiles. Should it be removed because it is not mentioned in materials?*

• Include a figure, like Fig. 2, with functional profiles per sample for the entire dataset.

Author’s response: *This would result in a very big figure with very low detail on individual samples, which would be a version of the Figure the referee wanted us to remove in the previous comment. We do not understand why this figure is needed. We are a bit puzzled with the referee’s comments.*

• “the most relevant features were extracted from the classification pipeline from each run of the experiment by averaging the feature importance of each base learner of the ensemble (an easily computable scores since we use decision trees)” so you used a threshold of a kind? Why is this not in the methods?.

Author’s response: *There is not a threshold for extracting relevant features. If you continue reading the text, the next sentence reads “The features that appeared in all the experiments were selected”. To make the text clearer we have changed the previous sentence for this one: “the most relevant features were extracted from the classification pipeline from each run of the experiment, cross referencing the nested loop for the best set of hyperparameters and a final fit with all training data, by averaging the feature importance of each base learner of the ensemble”.*

• You highlight the case of Ofa, but we don’t see the results for all other cities, so this is not useful. Just looking at NY tells us that there is much heterogeneity.

Author’s response: *As explained in the text, we commented only these results having a clear interpretation. The systematic interpretation of the results of all the cities is beyond the scope of a paper that just aim to demonstrate that functional profiles can be used for classification.*

• Section “Classification new samples of with new cities” – where are the results? The challenge was to predict cities from data, not to show PCA.

Author’s response: *CAMDA is an open-ended contest and, as we previously mentioned, we wanted to demonstrate that the functional profiles actually classify very well cities. We are not strictly following the challenge, which do not subtract novelty to our manuscript.*

• “Machine Learning Pipeline Comparison” – you don’t compare “pipelines” just the 3rd party tool that does the annotation. You have one pipeline. Revise.

Author’s response: *We have described three pipelines using KEGG, CARD and both (fusion) functional profiles in the text. We are comparing the classification accuracy in this section. Of course the functional annotation and the classification algorithms are 3rd party code: we do not want to reinvent the wheel. What is new here, as the title of the manuscript states, is the use of functional profiles for sample classification.*

• The goal of the challenge was to predict the mystery cities from the known cities, not use them as part of the training dataset. You can either do this and report the results, or do a “drop-one-city” analysis, where you calculate the prediction accuracy of predicting a certain city (you can calculate the average geographical distance of your predictor to that city) for all the samples in that city and repeat for all cities. These are your only predictive results. If you cannot do that then you have a classification algorithm and this should be made very clear.

Author’s response: *If the referee mean predict the name of an unseen mystery city, obviously neither our proposal nor other current algorithms with the samples given can predict the name of the city (maybe guessing that one of the mystery cities was Ilorin, close to Ofa. What we demonstrated is that new cities cluster apart, except in special cases such as Ofa-Ilorin or Auckland-Hamilton. What we also demonstrated by adding later the mystery cities samples and demonstrating the improvement of the predictor is that probably, the idea of the challenge of identifying new cities would become possible if the geography is more systematically sampled. We think the title of the manuscript and the text clarifies what we are proposing here.*

Minor issues

• From the abstract: “most likely origin of a sample” – what does that mean? You mean sampling site.

Author’s response: *Yes, it can be written in many different ways*.

• From the abstract: “provide an interesting functional point of view of the biogeography of the microbiota.” – most of the results were pretty similar, I fail to see a demonstration of any relationship. The case of Ofa is presented as an interesting point, but I cannot see how it can be generalized provided the diversity in NY, for example,

Author’s response: *We do not understand why the referee says that the results were pretty similar. Cities are separated by different sets of functional features (otherwise, they could have not been separated). In the case of Ofa the interpretation was easy, in the rest of cases it is beyond our skills and the scope of the manuscript. We only wanted to demonstrate that biologically relevant features can be used for the classification.*

• “we propose a machine learning innovative approach” -> “we propose an innovative machine learning approach”.

Author’s response: *Done.*

• Need more explanation on the KEGG/CARD. Was any threshold use? Each one offer multiple classifications for each gene, were they all used?.

Author’s response: *We have used here the MOCAT pipeline of the EMBL, one of the most widely used, which take all the functional labels for each gene.*

• Line 35, what is “CD-hit”?.

Author’s response: *The text reads “CD-hit [33]...” And, as the reference states, it is a computer application. We have clarified this in the text anyway.*

• Line 39, “a functional profile is generated for each sample by assessing the gene coverage” what does it mean “for each sample”? you wrote in line 37 that it is “for each city”? is the city-based classification used as a reference?.

Author’s response: *Each sample means exactly that: each sample is represented by a functional profile. In the text we explain that a gene catalog is created for each city. This is how functional annotation pipelines work.*

• The “Fusion pipeline” section is very unclear. How do you fuse the functional profiles? What latent space? A lot of buzzwords that tell me nothing on how this works and what you did. What do you mean “same response?” this is not a clinical database.

Author’s response: *As we explain in the text “feature fusion consists in concatenating, or adding, the latent representations of both views”.*

Buzzwords? Canonical Correlation Analysis is a known technique that reduces the space -latent space- (like, for example, PCA) and is described in the corresponding reference. The rest of words look quite extensively used (quantile, concatenating, features …). In addition to the explanation in the text, there is a reference to Fig. 1.

Same response = same result, output, tec. It is a common nomenclature. The word “response” is used in more domains than in clinic. Anyway, we have rephrased the sentence to “Given two datasets X1 and X2 that describe the same samples”.

• Figure 1B, doesn’t mention city profile and sample profile, at odds with what has been written above.

Author’s response: *As we mentioned before there are no city, but sample profiles. Cities are used to create gene catalogs.*

• Figure 1 is very helpful, but it should be clear form it how do we start with a sample and get a classification into a city (not prediction, as is currently stated).

Author’s response: Figure 1 explains the procedure used for training the predictor. Once the predictor is trained its use is obvious: it returns for a given functional profile the probability of belonging to a given city. As we have already commented, this is a predictor (generic) that classifies into city origins (specific task). See the functionality of the scikit-learn API used here: https://scikit-learn.org/stable/modules/generated/sklearn.ensemble.RandomForestClassifier.html#sklearn.ensemble.RandomForestClassifier.predict

• In the results section, “The CAMDA challenge” section is not a result, why does it need a separate section? You should embed it in the next section.

Author’s response: *Done*

• “in order to assert that the difference” – that’s not an assertion.

Author’s response: *It was a typo. We meant “assess”.*

• “The total number of extracted features adds up to 44.” – what features? Do you mean the functional profiles/categories? Why do you keep changing the terminology?

Author’s response: *We do not change the terminology. Actually, the title of the section is “Feature extraction and biological relevance in the classification”. In ML the variables, here the functional categories composing the profiles, are known as features. It is a well-known terminology.*

• “Importantly, the features used for the classification have a direct biological meaning and account” – repetitive.

Author’s response: *Why repetitive? We mentioned in the previous paragraph how to extract relevant features and here we state that the relevant features have a direct biological meaning.*

• I don’t understand the difference between Figs. 2 and 4. How did you convert the functional categories to a scale? Why Ofa, which in Fig. 2 looks like other cities, look different in Fig. 4.

Author’s response: *Figure legends explain what each figure is. There is no scale in* Fig. 2: *there are percentages of KEGG terms (collapsed to their highest-level category) found in the individual profiles of each population. This is not a peculiarity of Ofa. Ofa, like other cities, shows a distribution of high level KEGG terms relatively equivalent, but the predictor learns to distinguish among cities.*

• “Out of the seven most relevant features” – which 7 features? Where do I see them in Fig. 4?

Author’s response: *There is a blue square in the figure that clearly delimits 7 features (M0480 to M0257 from left to right in the X axis).*

• “Particularly, the Fluoroquinolone transport system (M00224) is” this should be in the discussion, it’s not a result.

Author’s response: *Please, note that the section is called “Results and discussion”.*

• “test the generalization power” there is no such thing generalization power." “obtained with the training dataset” – poor English. This whole paragraph is poorly written.

Author’s response: *OK, we have changed this for prediction power and rephrased the sentence*.

• “The accuracy of the predictor is of 0.73” – it is inappropriate to report accuracy in such manner. You should report the results in terms of specificity and sensitivity https://en.wikipedia.org/wiki/Sensitivity_and_specificity.

Author’s response: *We thank the wikipedia reference to specificity and sensitivity, we have learnt a lot. In any case, the idea here was to provide a general idea on the accuracy of the prediction. Since this is not the case of an unbalanced dataset or any anomalous scenario accuracy does the job very well. In any case, the confusion matrices in the* Tables 3 and 5 *provide specificity and sensitivity information.*

• “with no much biological interest” – poor English.

Author’s response: *Rephrased.*

• “Classification using different functional profiles” – move parts to the methods. Results section should consists of only/mainly results. “Although we do not have a strong” why here? This should be in the discussion.

Author’s response: *The subsection “Classification using different functional profiles” contains a discussion on why other profiles are interesting and results on the use of these profiles. It makes no sense moving it to Methods. Actually, in Methods, the functional profiles used are described in the subsection “Functional profiles”. And, please, note that the section is called “results and discussion” this is the reason why chunks of discussion follow to results.*

• “Since antibiotic resistance has emerged among the generic functionalities as a high relevant feature in the classification, in addition to have an obvious importance by itself, it seemed worth focusing on features that specifically describe antibiotic resistances.” I don’t see it.

Author’s response: *Well, there is a whole subsection called “Classification using different functional profiles” in which precisely we focus of antibiotic resistance profiles.*

• Consider merging Tables 5 and 3, graphically, not by content to reduce the number of tables.

Author’s response: *Mixing two confusion matrices would result into a confusing table. I have never seen this.*

• “Figure 6 shows the cities clustered as expected” – what was expected?

Author’s response: *It is expected that samples from the same city cluster together. We rephrased the sentence for better understanding.*

• “Thus, Ilorin and Ofa map together because these two cities are physically close cities in Nigeria (and connected by a train).” Really? they map together because they are physically close??? are you plotting them by distance?

Author’s response: *According to google maps only a train line links both cities and this line seem to have been sampled at both ends.*

• “As expected, the New York cluster shows the highest dispersion, although is not similar to Boston” – poor English.

Author’s response: *Rephrased*.

## Data Availability

Data sharing is not applicable to this article as no datasets were generated during the current study.

## Abbreviations

CAMDA: Critical Assessment of Massive Data Analysis
CARD: Comprehensive Antibiotic Resistance Database
CCA: Canonical Correlation Analysis
HLA: Human Leukocyte Antigen
KEGG: Kyoto Encyclopedia of Genes and Genomes
PCA: Principal Component Analysis
SNP: Single Nucleotide Polymorphisms
t-SNE: t-distributed Stochastic Neighbor Embedding
WGS: Whole genome sequencing
